# Supplementary material for: Cellular Environment Remodels the Genomic Fabrics of Functional Pathways in Astrocytes
Source: Genes (Basel). 2020 May 7;11(5):520. doi: 10.3390/genes11050520 (PMC7290327; doi:10.3390/genes11050520)
Supplement: Supplementary file 1 [file genes-11-00520-s001.pdf]

genes

Article

Oligodendrocytes remodel the genomic fabrics of functional pathways in astrocytes

Dumitru A Iacobas [1,2,*], Sanda Iacobas [3], Randy F Stout [4] and David C Spray [2,5]

Supplementary Material

Table S1. Genes whose >1.5x absolute fold-change did not meet the individual CUT criterion. Red/green background of the expression ratio indicates not significant (false) up-/down-regulation.

Gene	Description	X	CUT
Acap2	ArfGAP with coiled-coil, ankyrin repeat and PH domains 2	-1.540	1.816
Adamts18	a disintegrin-like and metallopeptidase	-1.514	1.594
Akr1c12	aldo-keto reductase family 1, member C12	1.866	1.994
Alx3	aristaless-like homeobox 3	1.536	1.769
Alyref2	Aly/REF export factor 2	-1.880	2.208
Ankrd33b	ankyrin repeat domain 33B	1.593	1.829
Ankrd45	ankyrin repeat domain 45	1.514	1.984
Ankrd50	ankyrin repeat domain 50	1.628	1.832
Ankrd61	ankyrin repeat domain 61	1.645	1.802
Arid1a	AT rich interactive domain 1A	-1.668	2.066
Artn	artemin	1.524	1.732
Aspm	abnormal spindle microtubule assembly	-1.693	1.716
Atp6v1e1	ATPase, H+ transporting, lysosomal V1 subunit E1	-1.679	1.777
Bag4	BCL2-associated athanogene 4	1.723	1.914
Birc3	baculoviral IAP repeat-containing 3	-1.588	1.722
Ccdc104	coiled-coil domain containing 104	-1.819	2.130
Ccl2	chemokine	-1.699	2.034
Cdc20b	cell division cycle 20 homolog B	1.512	1.605
Cenpf	centromere protein F	2.041	2.128
Cep97	centrosomal protein 97	-1.641	1.723
COX1	mitochondrially encoded cytochrome c oxidase I	-1.607	1.650
Cpsf7	cleavage and polyadenylation specific factor 7	-1.635	1.891
Crct1	cysteine-rich C-terminal 1	-1.530	1.701
Cul4b	cullin 4B	-1.550	1.574
Cyp4a12b	cytochrome P450, family 4, subfamily a, polypeptide 12B	-1.628	1.751
Ddx11	DEAD/H	2.057	2.228
Defb1	defensin beta 1	-1.581	1.629
Defb45	defensin beta 45	1.986	2.226
Deptor	DEP domain containing MTOR-interacting protein	1.839	1.899
Dmtf1	cyclin D binding myb-like transcription factor 1	-1.671	1.727
Dnaic1	dynein, axonemal, intermediate chain 1	-1.533	1.859
Dnmt3b	DNA methyltransferase 3B	-1.780	1.932
Draxin	dorsal inhibitory axon guidance protein	1.513	1.564
Eda2r	ectodysplasin A2 receptor	-1.525	1.682
Eif4g2	eukaryotic translation initiation factor 4, gamma 2	-2.150	2.324
Exoc5	exocyst complex component 5	-1.553	1.778
Fam110c	family with sequence similarity 110, member C	1.617	1.818
Fam124b	family with sequence similarity 124, member B	1.502	1.509
Fbxo34	F-box protein 34	-1.672	1.763
Fech	ferrochelatase	-1.624	1.908
Fgf18	fibroblast growth factor 18	1.716	1.729

Gdap10	ganglioside-induced differentiation-associated-protein 10	1.558	1.681
Gdf7	growth differentiation factor 7	1.501	1.560
Gfi1b	growth factor independent 1B	1.512	1.919
Glg1	golgi apparatus protein 1	-1.778	2.010
Gpr3	G-protein coupled receptor 3	-1.644	1.664
Gpx2	glutathione peroxidase 2	-1.527	1.794
Gria2	glutamate receptor, ionotropic, AMPA2	1.760	2.147
H2-Ke2	H2-K region expressed gene 2	-1.584	1.631
H2-Q4	histocompatibility 2, Q region locus 4	1.612	2.316
Herc6	hect domain and RLD 6	2.162	2.204
Hirip3	HIRA interacting protein 3	-1.636	1.877
Hmga2	high mobility group AT-hook 2	1.759	1.844
Hnrnpa2b1	heterogeneous nuclear ribonucleoprotein A2/B1	2.005	2.434
Hoxb6	homeobox B6	1.860	2.061
Hsd17b4	hydroxysteroid	-1.658	1.933
Id2	inhibitor of DNA binding 2	-1.647	1.665
Igh-VJ558	immunoglobulin heavy chain	-1.723	1.898
Il11	interleukin 11	-1.777	1.835
Isoc1	isochorismatase domain containing 1	-1.648	2.044
Itpripl2	inositol 1,4,5-triphosphate receptor interacting protein-like 2	-1.523	1.812
Kcna5	potassium voltage-gated channel, shaker-related subfamily, member 5	1.800	1.842
Kcnip1	Kv channel-interacting protein 1	-1.519	1.521
Klk11	kallikrein related-peptidase 11	-1.982	2.027
Kpna7	karyopherin alpha 7	-1.547	1.627
Krt6b	keratin 6B	1.647	1.826
Lamtor1	late endosomal/lysosomal adaptor, MAPK and MTOR activator 1	-1.726	1.745
Lrrc29	leucine rich repeat containing 29	1.696	1.845
Lrrc48	leucine rich repeat containing 48	2.168	2.295
Lrrc66	leucine rich repeat containing 66	1.705	2.131
Mad2l1bp	MAD2L1 binding protein	1.523	1.530
Megf10	multiple EGF-like-domains 10	-1.644	1.702
Mgat2	mannoside acetylglucosaminyltransferase 2	1.606	1.740
Mios	missing oocyte, meiosis regulator, homolog	1.653	1.669
Mpp5	membrane protein, palmitoylated 5	-1.551	1.908
Mrpl11	mitochondrial ribosomal protein L11	-1.562	2.125
Mrs2	MRS2 magnesium homeostasis factor homolog	-1.597	1.695
Mthfs	5, 10-methenyltetrahydrofolate synthetase	1.680	1.793
Mtss1l	metastasis suppressor 1-like	-1.580	1.669
Myo5b	myosin VB	-1.829	2.143
Nat8l	N-acetyltransferase 8-like	-1.661	2.099
Net1	neuroepithelial cell transforming gene 1	-1.686	2.039
Nfat5	nuclear factor of activated T cells 5	-1.740	1.791
Ngb	neuroglobin	1.561	1.665
Noc4l	nucleolar complex associated 4 homolog	1.697	1.948
Notch3	notch 3	-1.634	1.798
Nox4	NADPH oxidase 4	1.595	2.030
Olfr417	olfactory receptor 417	1.573	1.862
Pappa	pregnancy-associated plasma protein A	-1.608	2.168
Pcnt	pericentrin	-1.641	2.572
Pdf	peptide deformylase	-1.593	1.830
Pdxk	pyridoxal	-1.671	1.798
Pebp1	phosphatidylethanolamine binding protein 1	-1.517	1.852
Pias1	protein inhibitor of activated STAT 1	1.751	1.905
Pipox	pipecolic acid oxidase	-1.548	1.973
Plekhb1	pleckstrin homology domain containing, family B	1.514	1.566
Pm20d1	peptidase M20 domain containing 1	-1.538	1.665

Polr1d	polymerase	-1.707	1.914
Prr13	proline rich 13	1.908	1.915
Psg19	pregnancy specific glycoprotein 19	1.602	2.148
Pthlh	parathyroid hormone-like peptide	-1.545	1.905
Ptprn	protein tyrosine phosphatase, receptor type, N	1.849	1.955
Ptx4	pentraxin 4	1.571	1.667
Rffl	ring finger and FYVE like domain containing protein	1.900	1.988
Rhox3h	reproductive homeobox 3H	2.043	2.074
Rn18s	18S ribosomal RNA	1.838	1.998
Rnf139	ring finger protein 139	-1.728	1.754
Rpl19	ribosomal protein L19	-1.590	1.977
Rpl5	ribosomal protein L5	-1.604	2.085
Rplp2	ribosomal protein, large P2	-1.709	2.297
Rpp21	ribonuclease P 21 subunit	-1.678	1.720
Rtn4rl2	reticulon 4 receptor-like 2	1.614	1.816
Samd8	sterile alpha motif domain containing 8	1.536	1.758
Sec61b	Sec61 beta subunit	-1.683	1.812
Senp5	SUMO/sentrin specific peptidase 5	1.619	2.074
Serpinb3c	serine	-1.606	1.750
Sfn	stratifin	1.761	2.195
Slc38a11	solute carrier family 38, member 11	1.538	2.052
Slc6a5	solute carrier family 6	1.890	1.984
Smim5	small integral membrane protein 5	-1.850	2.290
Snord116	Pwcr1 mRNA, complete sequence.	2.012	2.167
Snrpg	small nuclear ribonucleoprotein polypeptide G	-1.554	1.926
Stmn4	stathmin-like 4	1.546	1.679
Stpg2	sperm tail PG rich repeat containing 2	1.628	2.123
Syt1	synaptotagmin I	1.992	2.153
Syt6	synaptotagmin VI	1.670	1.671
Tal1	T cell acute lymphocytic leukemia 1	1.660	1.763
Tbccd1	TBCC domain containing 1	-1.509	1.688
Thoc3	THO complex 3	-1.643	2.254
Thoc5	THO complex 5	-1.582	1.666
Tinagl1	tubulointerstitial nephritis antigen-like 1	-1.585	1.676
Tm9sf1	transmembrane 9 superfamily member 1	-1.664	2.007
Tmem201	transmembrane protein 201	1.732	2.208
Tmem242	transmembrane protein 242	-1.592	1.721
Tspan32	tetraspanin 32	1.562	1.833
Ubl3	ubiquitin-like 3	-1.521	1.786
Vpreb3	pre-B lymphocyte gene 3	-1.692	1.709
Zbed3	zinc finger, BED domain containing 3	1.615	1.643
Zc3h14	zinc finger CCCH type containing 14	-1.850	2.064
Zfml	zinc finger, matrin-like	-1.694	1.794
Zfp235	zinc finger protein 235	1.776	1.821
Zfp354b	zinc finger protein 354B	-1.883	1.901
Zkscan5	zinc finger with KRAB and SCAN domains 5	-1.671	1.834

Table S2. Genes considered as significantly up-regulated although their absolute fold-change was below the traditional 1.5x.

Gene	Description	X	CUT
Adora2b	adenosine A2b receptor	1.441	1.194
Anapc2	anaphase promoting complex subunit 2	1.230	1.087
Arrb1	arrestin, beta 1	1.167	1.138
Arrb2	arrestin, beta 2	1.264	1.216
Atg2a	autophagy related 2A	1.173	1.160
Ccl5	chemokine	1.488	1.371
Cd37	CD37 antigen	1.413	1.153
Cd38	CD38 antigen	1.335	1.224
Chuk	conserved helix-loop-helix ubiquitous kinase	1.100	1.097
Col6a1	collagen, type VI, alpha 1	1.258	1.217
Col9a2	collagen, type IX, alpha 2	1.239	1.233
Ctsb	cathepsin B	1.460	1.250
Ednrb	endothelin receptor type B	1.428	1.245
Eif2s1	eukaryotic translation initiation factor 2, subunit 1 alpha	1.274	1.242
Fgf1	fibroblast growth factor 1	1.442	1.218
Fgf9	fibroblast growth factor 9	1.498	1.250
Fgr	Gardner-Rasheed feline sarcoma viral	1.330	1.269
Foxo1	forkhead box O1	1.401	1.107
G6pc3	glucose 6 phosphatase, catalytic, 3	1.212	1.163
Glul	glutamate-ammonia ligase	1.433	1.260
Gnal	guanine nucleotide binding protein, alpha stimulating, olfactory type	1.337	1.329
Gng11	guanine nucleotide binding protein	1.195	1.192
Gng3	guanine nucleotide binding protein	1.443	1.254
Gsn	gelsolin	1.269	1.267
Gucy1b2	guanylate cyclase 1, soluble, beta 2	1.346	1.268
Igbp1	immunoglobulin	1.131	1.130
Igf1	insulin-like growth factor 1	1.330	1.245
Itga9	integrin alpha 9	1.299	1.193
Itgb2	integrin beta 2	1.403	1.155
Jak1	Janus kinase 1	1.143	1.135
Limk1	LIM-domain containing, protein kinase	1.289	1.239
Mad2l2	MAD2 mitotic arrest deficient-like 2	1.443	1.335
Mapk3	mitogen-activated protein kinase 3	1.379	1.324
Med24	mediator complex subunit 24	1.260	1.242
Naip2	NLR family, apoptosis inhibitory protein 2	1.249	1.203
Ncf1	neutrophil cytosolic factor 1	1.325	1.183
Nod1	nucleotide-binding oligomerization domain containing 1	1.327	1.196
Nos3	nitric oxide synthase 3, endothelial cell	1.274	1.225
Panx1	pannexin 1	1.423	1.138
Pde1b	phosphodiesterase 1B, Ca2+-calmodulin dependent	1.402	1.145
Phka2	phosphorylase kinase alpha 2	1.165	1.090
Pik3r5	phosphoinositide-3-kinase, regulatory subunit 5, p101	1.345	1.179
Pik3r6	phosphoinositide-3-kinase, regulatory subunit 6	1.093	1.092
Pip4k2c	phosphatidylinositol-5-phosphate 4-kinase, type II, gamma	1.131	1.079
Rac3	RAS-related C3 botulinum substrate 3	1.262	1.170
Rbck1	RanBP-type and C3HC4-type zinc finger containing 1	1.228	1.122
Slc2a1	solute carrier family 2	1.497	1.408
Sphk2	sphingosine kinase 2	1.179	1.117
Ssh3	slingshot homolog 3	1.309	1.161
Syk	spleen tyrosine kinase	1.219	1.131
Tab1	TGF-beta activated kinase 1/MAP3K7 binding protein 1	1.249	1.056
Tbc1d4	TBC1 domain family, member 4	1.392	1.365

Tgfb1	transforming growth factor, beta 1	1.372	1.311
Ticam1	toll-like receptor adaptor molecule 1	1.285	1.246
Tsc2	tuberous sclerosis 2	1.222	1.218
Txnip	thioredoxin interacting protein	1.304	1.250

Table S3. Genes considered as significantly down-regulated although their absolute fold-change was below the traditional 1.5x.

Gene	Description	X	CUT
Adcy2	adenylate cyclase 2	-1.267	1.214
Adrb3	adrenergic receptor, beta 3	-1.437	1.432
Antxr2	anthrax toxin receptor 2	-1.340	1.232
Arhgef6	Rac/Cdc42 guanine nucleotide exchange factor	-1.384	1.200
Atg16l2	autophagy related 16-like 2	-1.485	1.242
Bcar1	breast cancer anti-estrogen resistance 1	-1.438	1.229
Bcl2l1	BCL2-like 1	-1.395	1.242
Bdnf	brain derived neurotrophic factor	-1.488	1.221
Braf	Braf transforming gene	-1.241	1.209
Bub1	budding uninhibited by benzimidazoles 1 homolog	-1.470	1.362
Camk2d	calcium/calmodulin-dependent protein kinase II, delta	-1.182	1.152
Casp1	caspase 1	-1.388	1.231
Ccna2	cyclin A2	-1.348	1.195
Cdc14b	CDC14 cell division cycle 14B	-1.231	1.221
Cdc20	cell division cycle 20	-1.311	1.166
Cdc25a	cell division cycle 25A	-1.227	1.226
Cdk2	cyclin-dependent kinase 2	-1.482	1.226
Cdk6	cyclin-dependent kinase 6	-1.338	1.228
Cfl2	cofilin 2, muscle	-1.355	1.287
Col1a1	collagen, type I, alpha 1	-1.497	1.455
Col1a2	collagen, type I, alpha 2	-1.318	1.176
Col2a1	collagen, type II, alpha 1	-1.281	1.128
Creb3l4	cAMP responsive element binding protein 3-like 4	-1.267	1.256
Cry1	cryptochrome 1	-1.227	1.198
Ctnnb1	catenin	-1.305	1.285
Cxcr3	chemokine	-1.375	1.134
E2f1	E2F transcription factor 1	-1.255	1.168
Eif2ak3	eukaryotic translation initiation factor 2 alpha kinase 3	-1.444	1.269
Fadd	Fas (TNFRSF6)-associated via death domain	-1.123	1.115
Fgd3	FYVE, RhoGEF and PH domain containing 3	-1.244	1.209
Flt3l	FMS-like tyrosine kinase 3 ligand	-1.286	1.275
Gadd45a	growth arrest and DNA-damage-inducible 45 alpha	-1.342	1.190
Gadd45b	growth arrest and DNA-damage-inducible 45 beta	-1.378	1.223
Gbp5	guanylate binding protein 5	-1.335	1.326
Gjc1	gap junction protein, gamma 1	-1.316	1.142
Gnaq	guanine nucleotide binding protein, alpha q polypeptide	-1.284	1.220
Gng12	guanine nucleotide binding protein	-1.282	1.161
Gng2	guanine nucleotide binding protein	-1.134	1.099
Hif1a	hypoxia inducible factor 1, alpha subunit	-1.361	1.235
Hsp90b1	heat shock protein 90, beta	-1.183	1.145
Ins2	insulin II	-1.334	1.194
Insr	insulin receptor	-1.337	1.315
Iqgap1	IQ motif containing GTPase activating protein 1	-1.416	1.183
Iqgap2	IQ motif containing GTPase activating protein 2	-1.391	1.248
Iqgap3	IQ motif containing GTPase activating protein 3	-1.478	1.316
Itgav	integrin alpha V	-1.434	1.294
Itgb5	integrin beta 5	-1.368	1.160

Itpr1	inositol 1,4,5-trisphosphate receptor 1	-1.338	1.127
Lama2	laminin, alpha 2	-1.438	1.255
Mad2l1	MAD2 mitotic arrest deficient-like 1	-1.484	1.393
Mapk12	mitogen-activated protein kinase 12	-1.350	1.324
Mapk8	mitogen-activated protein kinase 8	-1.356	1.317
Mcl1	myeloid cell leukemia sequence 1	-1.166	1.123
Mcm2	minichromosome maintenance deficient 2 mitotin	-1.399	1.304
Mcm4	minichromosome maintenance deficient 4 homolog	-1.398	1.185
Mcm7	minichromosome maintenance deficient 7	-1.320	1.229
Mdm2	transformed mouse 3T3 cell double minute 2	-1.301	1.270
Med30	mediator complex subunit 30	-1.199	1.164
Msn	moesin	-1.330	1.137
Myl2	myosin, light polypeptide 2, regulatory, cardiac, slow	-1.386	1.361
Orc2	origin recognition complex, subunit 2	-1.273	1.212
Orc6	origin recognition complex, subunit 6	-1.195	1.171
P2ry1	purinergic receptor P2Y, G-protein coupled 1	-1.213	1.119
Pak3	p21 protein	-1.350	1.189
Pdgfc	platelet-derived growth factor, C polypeptide	-1.328	1.114
Per2	period circadian clock 2	-1.372	1.232
Pfn1	profilin 1	-1.342	1.341
Phlpp1	PH domain and leucine rich repeat protein phosphatase 1	-1.144	1.111
Pkn3	protein kinase N3	-1.341	1.185
Ppp1r12a	protein phosphatase 1, regulatory	-1.292	1.288
Prkaa2	protein kinase, AMP-activated, alpha 2 catalytic subunit	-1.499	1.190
Rad21	RAD21 homolog	-1.340	1.276
Rb1cc1	RB1-inducible coiled-coil 1	-1.233	1.129
Rbl1	retinoblastoma-like 1	-1.470	1.362
Rcan1	regulator of calcineurin 1	-1.246	1.184
Rock1	Rho-associated coiled-coil containing protein kinase 1	-1.263	1.237
Rock2	Rho-associated coiled-coil containing protein kinase 2	-1.329	1.311
Shc3	src homology 2 domain-containing transforming protein C3	-1.307	1.227
Slc16a10	solute carrier family 16	-1.320	1.215
Smad2	SMAD family member 2	-1.205	1.134
Smc1a	structural maintenance of chromosomes 1A	-1.344	1.239
Spp1	secreted phosphoprotein 1	-1.448	1.406
Stat2	signal transducer and activator of transcription 2	-1.463	1.273
Tbk1	TANK-binding kinase 1	-1.332	1.225
Tgfb3	transforming growth factor, beta 3	-1.394	1.199
Thbs3	thrombospondin 3	-1.321	1.096
Thrb	thyroid hormone receptor beta	-1.361	1.221
Trpc1	transient receptor potential cation channel, subfamily C, member 1	-1.221	1.188
Trpv2	transient receptor potential cation channel, subfamily V, member 2	-1.215	1.101
Tsc1	tuberous sclerosis 1	-1.185	1.164
Uvrag	UV radiation resistance associated gene	-1.416	1.245
Vav3	vav 3 oncogene	-1.234	1.225
Vegfc	vascular endothelial growth factor C	-1.307	1.089
Wee1	WEE 1 homolog 1	-1.195	1.124
Ywhah	tyrosine 3-monooxygenase/tryptophan 5-monooxygenase activation protein, eta polypeptide	-1.360	1.307

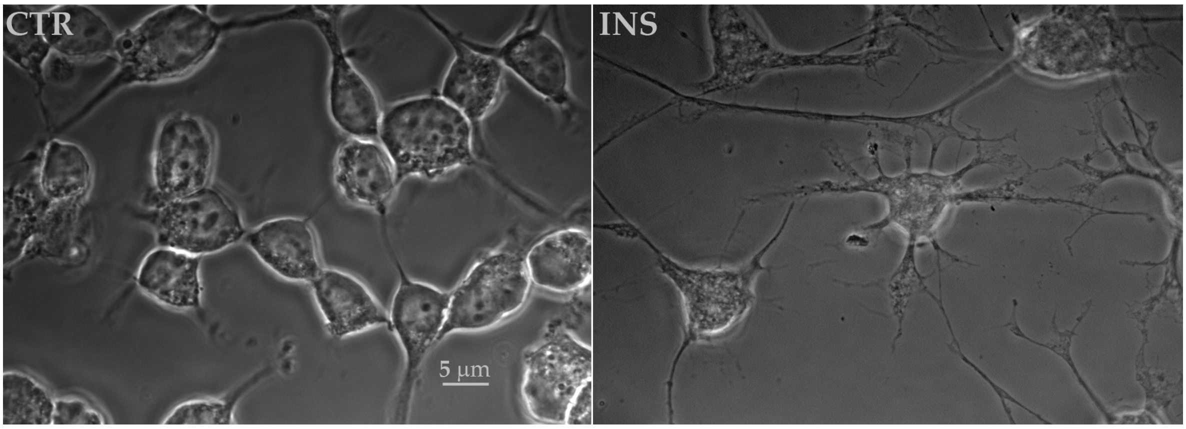

Figure S1. MAGlabeled phase contrast micrographs showing the morphological changes of Oli-neu cells cultured alone (CTR) and co-cultured with astrocytes (INS). **Note the progression from precursor oligodendrocytes to preoligodendrocytes.**

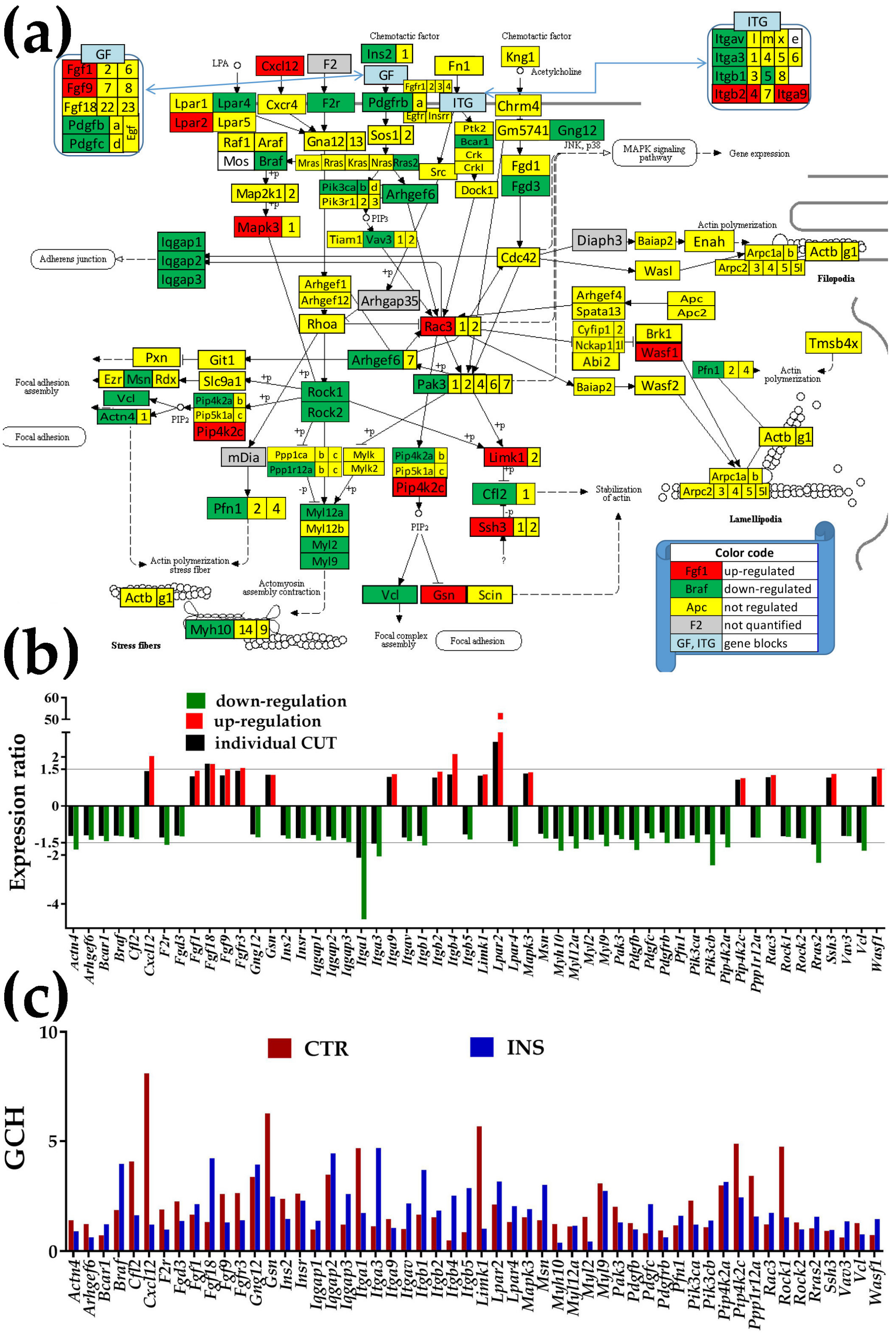

Figure S2. Presence of non-touching precursor oligodendrocytes regulate the actin cytoskeleton (AC) pathway. **(a)** Regulation of interconnected genes with the AC pathway. **(b)** Expression ratios and individual fold-change cut-offs (negative for down-regulation) of the significantly regulated genes. Note that regulation of several genes would be neglected by the traditional analysis, while for others the individual gene cut-off exceeded 1.5x. **(c)** Oli-neu proximity changes the gene hierarchy. The display is limited to the significantly regulated genes plus the actins: *Actb*, *Actg1* and *Actn1*. The dominant gene in this pathway is *Pip4k2c* with GCH = 15.00 in INS.

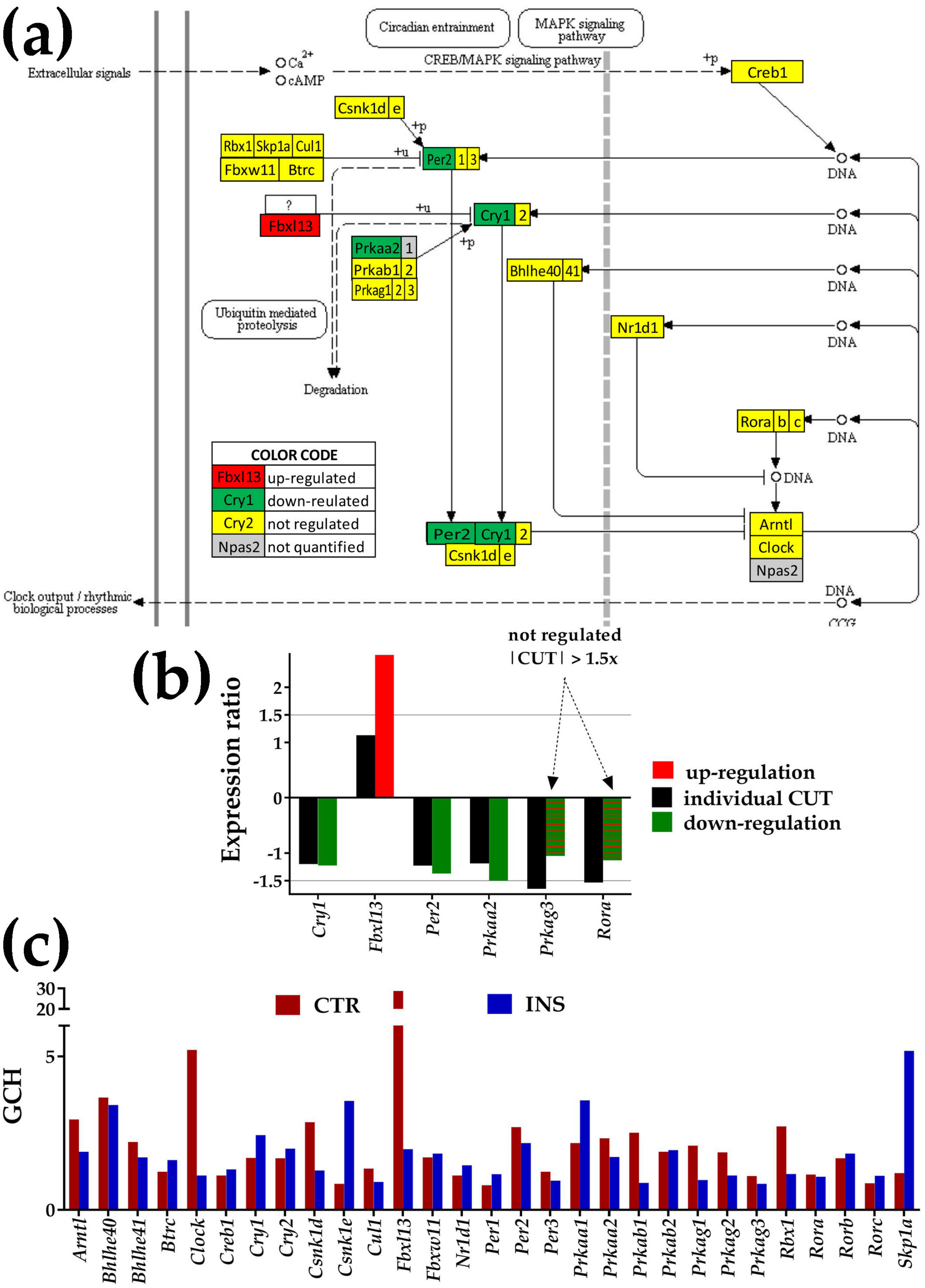

Figure S3. Presence of non-touching precursor oligodendrocytes regulates the Circadian Rhythm. (CR) pathway. **(a)** Regulation of the interconnected genes within CC pathway. **(b)** Expression ratios and individual fold-change cut-offs (negative for down-regulation) of the significantly regulated genes. Note that regulation of *Cry1* and *Per22* would be neglected by the traditional analysis and that the individual gene cut-off for *Prkag3* and *Rora* exceeded 1.5x. **(c)** Changes in the gene commanding height (GCH) scores. The most prominent gene, towering by far all other genes in the CTR astrocytes is *Fbxl13* (GCH = 28.82).
